# Participation in the International Judo Federation World Tour competitions and performance in Tokyo Olympic Games

**DOI:** 10.3389/fspor.2023.1216002

**Published:** 2023-06-27

**Authors:** Danilo F. C. Santos, Rafael L. Kons, João P. Lopes-Silva, Marcus F. Agostinho, Daniele Detanico, Monica Y. Takito, Emerson Franchini

**Affiliations:** ^1^ Martial Arts and Combat Sports Research Group, Sport Department, School of Physical Education and Sport, University of São Paulo, São Paulo, Brazil; ^2^ Department of Physical Education, Federal University of Bahia, Salvador, Bahia, Brazil; ^3^ Applied Research Group to Performance and Health, CESMAC University Center, Maceió, Alagoas, Brazil; ^4^ Biomechanics Laboratory, Sports Centre, Federal University of Santa Catarina, Santa Catarina, Brazil; ^5^ Human Movement Pedagogy, School of Physical Education and Sport, University of São Paulo, São Paulo, Brazil

**Keywords:** world-ranking, medals, long-term performance, high-level competitions, performance prediction

## Abstract

**Introduction:**

The International Judo Federation introduced a ranking system in 2009 that determines top athletes for the Olympic Games and seeds them in competitions. Previous research indicated that this ranking list and past performances predicted 19%–27% of performance in the Olympic Games and World Championships. However, due to the COVID-19 pandemic, the relationship between Judo World Tour competitions and Olympic Games performance may have been affected. This study aimed to examine the relationship between athletes' performance in Judo World Tour competitions and their competitive performance at the Olympic Games.

**Methods:**

Data from 393 athletes who participated in the Tokyo Olympics were analyzed considering both long and short-term performance measures. Pearson correlation was used to determine the relationship between variables and multiple linear regressions were used to predict performance for each sex and the entire sample.

**Results:**

The results revealed a range of magnitudes in the correlation between variables, varying from small to large. In terms of regression analyses, it was observed that, for females, the percentage of matches won during the classification period and competition in the year prior to the Olympic Games predicted 37% of their performance. For males, the percentage of matches won during the classification period and competition in the six months before the Olympic Games predicted 36% of their performance.

**Discussion:**

Thus, athletes’ quality and reduced exposure to competition near the Olympic Games appear to be important factors in their performance at the event.

## 1. Introduction

To qualify to the Olympic Games, the International Judo Federation (IJF) established a classification based on a world ranking list in 2009 (1). This ranking is based on performance in different competition types, such as Continental Open, Continentals, Grand Prix, Grand Slam, World Master, World Championship, and Olympic Games. Each of these competitions has a specific number of points associated with it: 100 for the Continental Open, 700 for the Continental Championship, 700 for the Grand Prix, 1000 for the Grand Slams, 1800 for the World Masters and 2000 for the World Championships. The silver and bronze medal performances represent 70% and 50% of the points, respectively. Additionally, smaller amounts of points are given for fifth, seventh, and other positions, as well as for participation. To qualify for the Olympic Games, the top six results in each of the two previous years are considered, and the points obtained in the 13th to the 24th months have 50% of their original value, while in 100% of the points obtained in the last year are considered. During this period, athletes participate in Grand Slams, Grand Prix, and World Cups. Furthermore, the top eight ranked athletes are seeded in draws to avoid the best athletes competing against each other in the elimination phase (2).

Several investigations have examined the impact of ranking position on Olympic Games medal distribution of judo athletes (3–6). It was found that variables from the World Ranking List, including points valid in the two-year period and number of competitions in 2012, could predict 24% (for females) and 26% (for males) of judo performance during London 2012 Olympic Games (3). Additionally, the performance of judo athletes in the World Championships has been examined across different age categories, including cadet, junior, and senior (5). They reported that the World Ranking List position and competitive performance during the year of the World Championships, measured by placing at competitions, could predict between 5% and 27% of the athletes' performance. Studies indicated that during the London Olympic Games, the top eight ranked athletes gained most of the medals distributed, and that there was a probability of seeded male athletes winning a medal of 41.1% and 42.9% in London 2012 and Rio 2016 Olympic Games, respectively (4), while for females, the probabilities were 35.7% and 44.6% in London 2012 and Rio 2016, respectively (4). More recently (6), it was demonstrated that seeded athletes (the top eight ranked athletes) have about a 15% higher chance of winning a medal and a 10% higher chance of reaching the quarterfinals when considering professional judo competitions. Additionally, the first and second seeded athletes have an advantage in winning the gold medal. Specifically, this author indicated that world ranking position and performance in the classification phase could predict the judo athletes’ performance in the major events such as World Championship and Olympic Games.

Unlike the situations in London 2012 and Rio 2016, the classification phase for Tokyo 2020 was different due to the COVID-19 pandemic (7). Competitions that were scheduled during the classification phase were cancelled, and when competitions resumed, they had short intervals between them (range of 4–30 days for the Judo World Tour and 48 days between Word Championship and Olympic Games). This resulted in the classification period finishing too close to the Olympic Games, which may have been a problem for athletes, since longer intervals have previously been reported to be associated with increased performance during the Olympic Games (8). For the first time, the Judo World Championship was disputed in the same year of the Olympic Games, with only 48 days separating these competitions (9). As a result, athletes faced two main scenarios: (1) compete in the World Championship to avoid their direct competitors from taking their place and qualify to the Olympic Games despite a very short interval between two top judo competitions, or (2) do not participate in the World Championship, have a longer interval between their last competition and the Olympic Games, and count on the fact that their direct competitors for a place would not qualify, or (3) go for the ultimate glory and try to get medals in both competitions.

Results from a recent study showed that athletes who participated in the World Championships and won medals had a 12.7% higher chance of winning a medal in the Olympic Games compared to non-medalists (9). On the other hand, athletes who did not participate in the World Championships also had an increased odds ratio (6.6) of winning an Olympic medal compared to those who participated in the World Championships but did not win a medal (9). Therefore, given the COVID-19 pandemic during the qualification period, which affected the training process of many athletes (10), the two most important judo tournaments were conducted in less than 48 days (9), and a higher number of competitions composed the ranking system with different point attribution, a different association could be found between performance in the Judo World Tour competitions and performance in the Olympic Games than those reported in previous studies (3, 6). Thus, this investigation aimed to verify the relationship between performance in the Judo World Tour competitions (i.e., those used to determine the IJF ranking) and athletes' performance during the Tokyo 2020 Olympic Games. The main hypothesis of the present study was that athletes better positioned in the ranking, but with a longer interval between competitions, would perform better in the Olympic Games.

## 2. Method

### 2.1. Research design

This study is a descriptive analysis of the Tokyo 2020 Olympic Games, focusing on the athletes' performance in the Olympics while considering their previous performance in the World Tour competitions.

### 2.2. Participants

Data from 393 athletes who took part in the Olympic Games were analyzed. Of these athletes, six who did not participate in the World Tour competition were invited by the IJF to participate in the Olympic Games. Therefore, there was no sampling process, as all athletes who participated in the Olympic Games were analyzed. Table 1 presents the number of athletes for each weight category during the Tokyo 2020 Olympic Games. The data from results of Tokyo Olympic Games was obtained from publicly available online sources (https://judobase.ijf.org). In addition, informed consent was not required for the study, according to the Belmont Report (NH, 1978) and according to the approval of the ethics committee of University of Sao Paulo (number: 5.920.124). The study was conducted in accordance with the Declaration of Helsinki. The official results books were documented by a judo expert referee of the International Judo Federation. The data for the independent variables were obtained from the International Judo Federation Website (https://judobase.ijf.org).

**Table 1:** Number and percentage (in parentheses) of athletes who participated in judo competition during the Tokyo 2020 Olympic Games.

Weight Categories	Male	Female
Extra-lightweight (M = ≤60 kg; F = ≤48 kg)	23 (11)	28 (15)
Half-lightweight (M = ≤66 kg; F = ≤52 kg)	27 (13)	29 (15)
Lightweight (M = ≤73 kg; F = ≤57 kg)	36 (18)	25 (13)
Half-middleweight (M = ≤81 kg; F = ≤63 kg)	35 (17)	31 (16)
Middleweight (M = ≤90 kg; F = ≤70 kg)	33 (16)	28 (15)
Half-heavyweight (M = ≤100 kg; F = ≤78 kg)	25 (12)	24 (13)
Heavyweight (M = >100 kg; F = >78 kg)	22 (11)	27 (14)
Total	201 (51)	192 (49)

M, male; F, female.

### 2.3. Procedures

The dependent variable was athletes' performance at the Olympic Games. To measure this variable, we recorded the competition results (1st, 2nd, 3rd, 5th, 7th), and used the IJF points for each placing. Points at the Olympic Games varied as follows: Gold medal = 2,200; silver medal = 1,540; bronze medal = 1,100; 5th place = 792; 7th place = 572; 16th = 352; 32nd = 264.

The independent variables were: (1) Measures of athletes' performance in the two years before the Olympic Games (athletes' performance during the entire classification phase, which was held between 25th May 2018 to 28th June 2021, i.e., points accumulated during this period); (2) Final position in the IJF ranking list, excluding athletes from the same country; (3) Measures of athletes' performance during phase with 100% of the points considered for Olympic Games classification occurred one year and six months before the Tokyo Olympic Games (i.e., between 24th May 2019 and 28th June 2021); (4) number of matches won and lost two years, one year, and six months before the Olympic Games; (5) Number of competition disputed two years, one year, and six months before the Olympic Games; (6) Interval (in days) between the last competition and the Olympic Games.

### 2.4. Statistical analysis

The Levene's test was used to test for homogeneity and the data normality was accessed by Kolmogorov-Smirnov, and all data were normally distributed. As the data was found to be homogeneous, Pearson correlation was used to determine the relationship between variables. Pairwise multiple linear regressions were conducted to predict performance in the Tokyo Olympics for each sex and for the entire sample. Specifically, the Stepwise method was used. Collinearity analysis was also performed, where variables that exhibited significant correlation (*p* < 0.05), variables with a variance inflation factor (VIF) ≥ 5, tolerance ≤0.1, and Durbin-Watson index outside the range of 2 were not included simultaneously in the model. All analyses were performed separately for female and male athletes. The descriptive results were presented as the coefficient of correlation value (*R*), the coefficient of determination value (*R*^2^), the adjusted *R*^2^ value, the standard error of estimate value (SEE), and the significance level (*P*). The magnitude of correlation was classified according to Hopkins (11): *r* = 0–0.1 (trivial), 0.11–0.3 (small), 0.31–0.5 (moderate), 0.51–0.7 (large), 0.71–0.9 (very large), and 0.91–1.0 (almost perfect). The significance level was set at 5% for all analyses, and all analyses were conducted using Statistica for Windows, version 12 (StatSoft, Tulsa, United States of America).

## 3. Results

**Figure 1:**
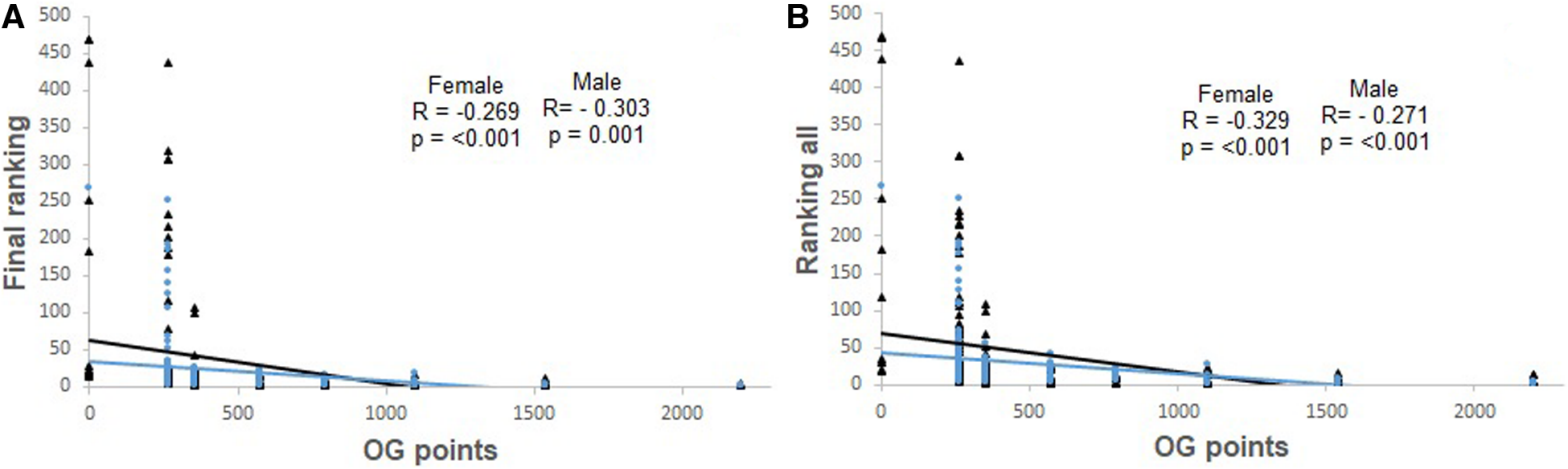
Correlation between Final Ranking (after discarding for Olympic Games classification) and Olympic Games points (OG points) (Panel **A**), and between Final Ranking all (without discarding) and Olympic Games points (OG points) (Panel **B**). The analyses include male (represented by black triangles and black lines) and female athletes (represented by blue circles and blue lines).

**Figure 2:**
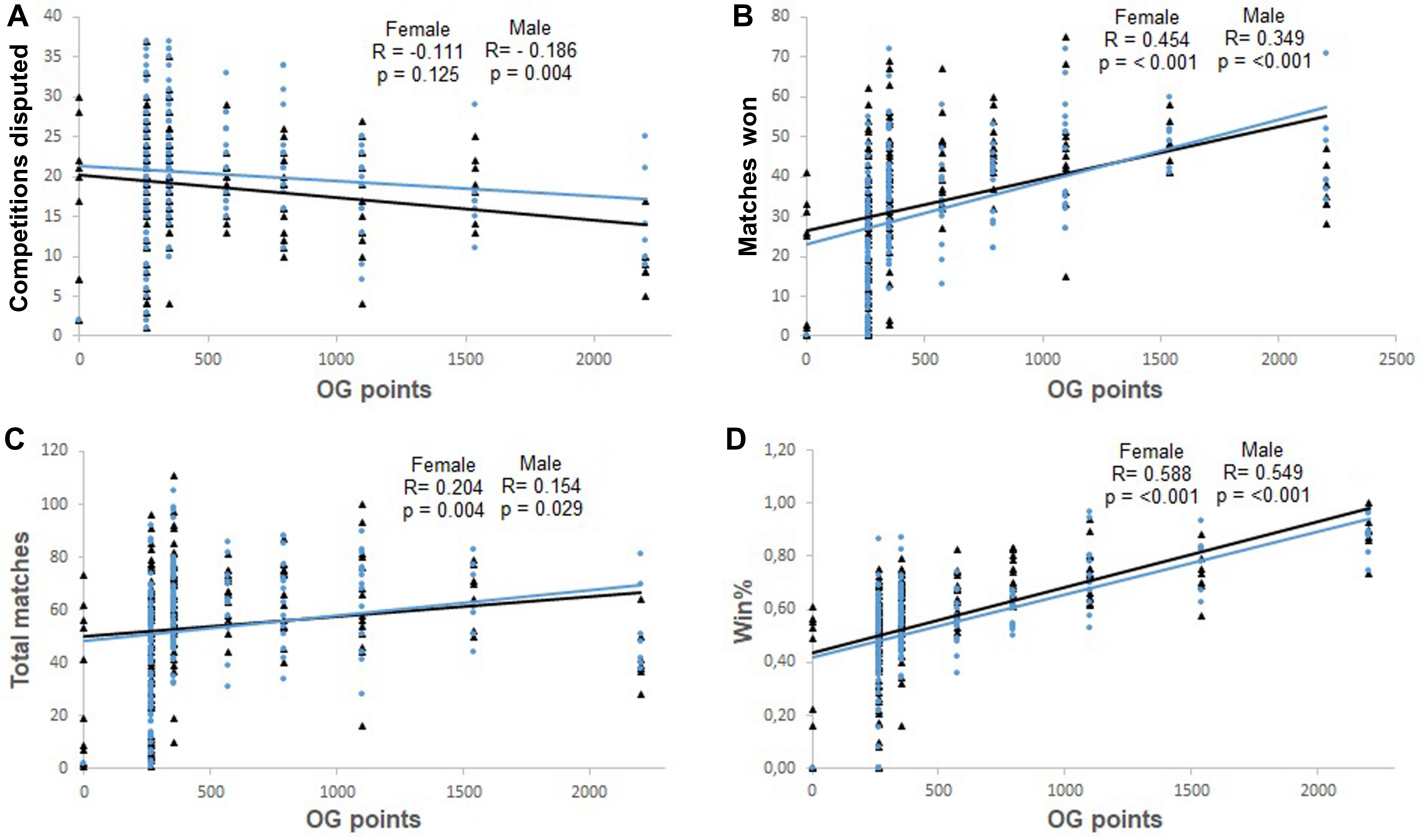
Correlation between the number of competitions contested in the classification phase and Olympic Games points (OG points) (Panel **A**), between matches won in the classification phase and Olympic Games points (OG points) (Panel **B**), between the total number of matches in the classification phase and Olympic Games points (OG points) (Panel **C**), and between the winning percentage (Win %) during the classification phase and Olympic Games points (OG points) (Panel **D**). The analyses include male (represented by black triangles and black lines) and female athletes (represented by blue circles and blue lines).

**Figure 3:**
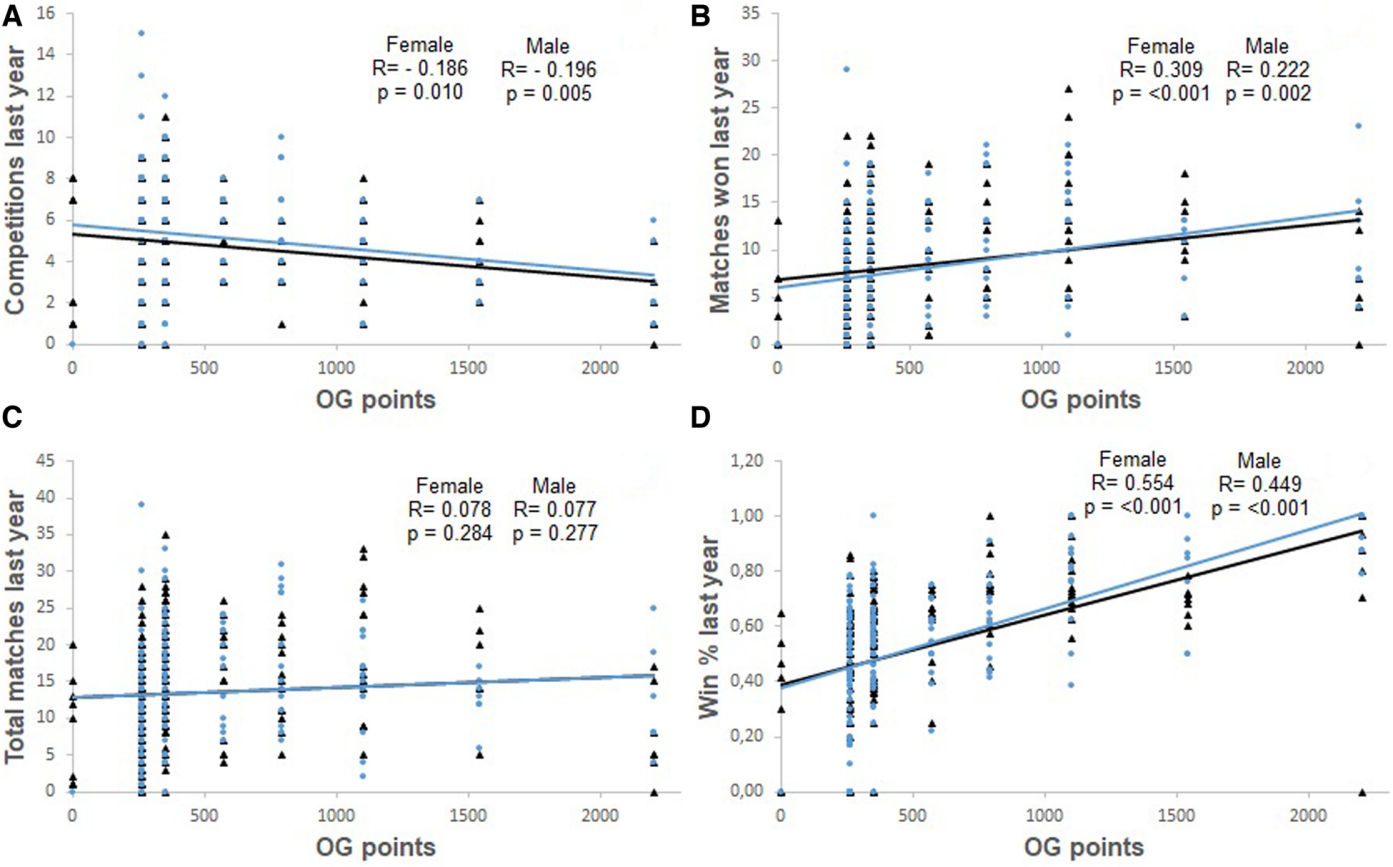
Correlation between the number of competitions contested one year before the Olympic Games and Olympic Games points (OG points) (Panel **A**), between matches won during the competition one year before the Olympic Games and Olympic Games points (OG points) (Panel **B**), between the total number of matches contested one year before the Olympic Games and Olympic Games points (OG points) (Panel **C**), and between the winning percentage one year before the Olympic Games (Win % last year) and Olympic Games points (OG points) (Panel **D**). The analyses include male (represented by black triangles and black lines) and female athletes (represented by blue circles and blue lines).

**Figure 4:**
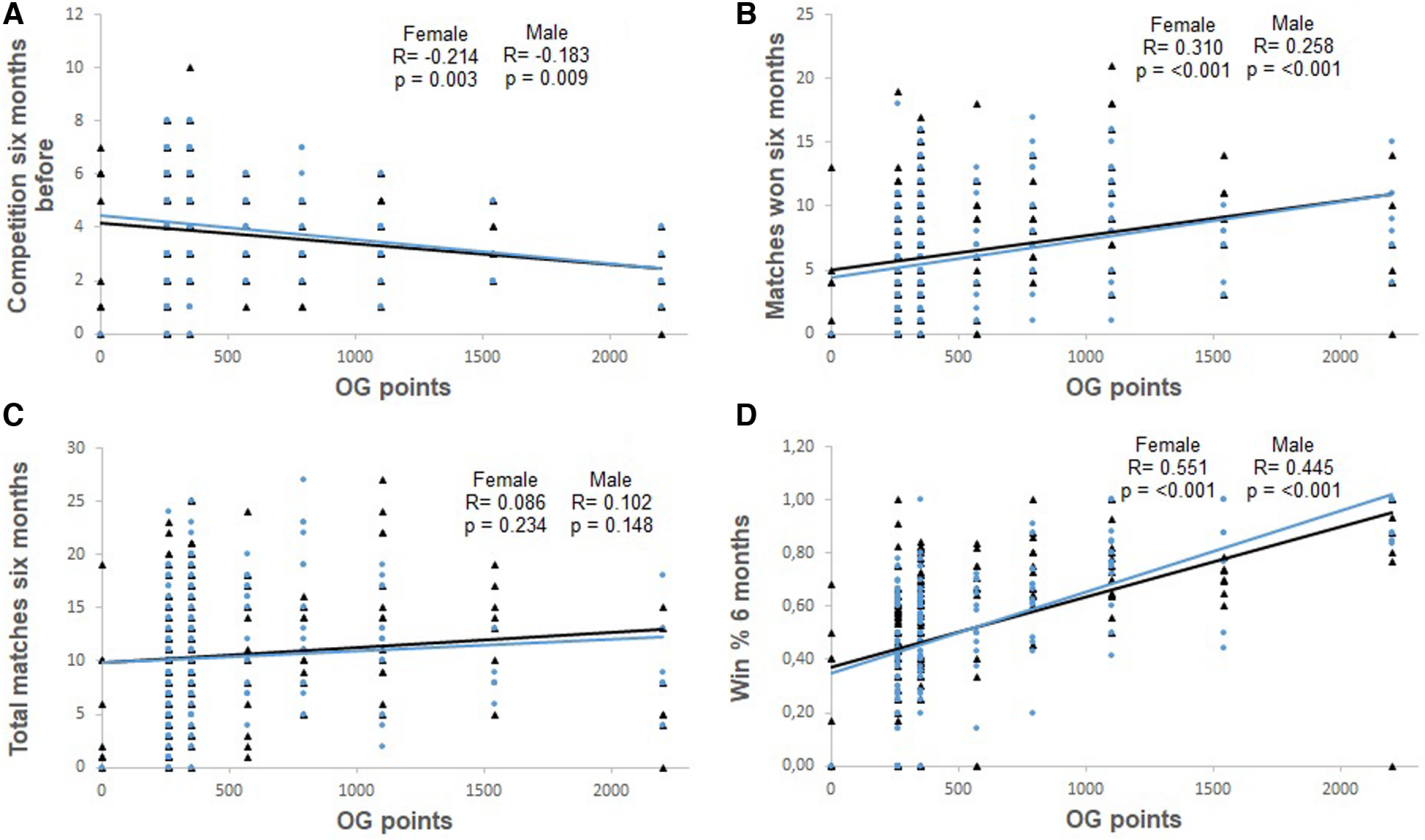
Correlation between the number of competitions contested six months before the Olympic Games and Olympic Games points (OG points) (Panel **A**), between matches won during the competition six months before the Olympic Games and Olympic Games points (OG points) (Panel **B**), between the total number of matches contested during the six months before the Olympic Games and Olympic Games points (OG points) (Panel **C**), and between the winning percentage in the six months before the Olympic Games (Win % 6 months) and Olympic Games points (OG points) (Panel **D**). The analyses include male (represented by black triangles and black lines) and female athletes (represented by blue circles and blue lines).

Figures 1–4, presents the relationship between Olympic Games performance and the variables of previous performance. In addition, the relationship between Olympic Games and variables based on the World Ranking list and previous performance in each female and male weight category, and all females and males grouped is present in Table 2. The magnitude of the correlation between variables varied from small to large.

**Table 2:** Relationship (and r-value 95% confidence intervals) between performance in the Olympic games and variables based on the world ranking list and previous performance in each female and male weight category, and all females and males grouped.

	Final ranking	Ranking all	Competition disputed	Matches won	Total matches	Win%	Competitions last year	Matches won last year	Total matches last year	Win% last year	Competition 6 months	Matches won 6 months	Total matches 6 months	Win% 6 months
OG points Female (≤48 kg) *n* = 28	*r* = −0.304 (−0.613 −0.086) (moderate); *p* = 0.123	*r* = −0.397 (−0.671 −0.028) (moderate); *p* = 0.037	*r* = −0.082 (−0.441 0.300) (trivial); *p* = 0.679	*r* = 0.540 (0.209 0.760) (large) *p* = 0.003	*r* = 0.224 (−0.162 0.551) (small); *p* = 0.251	*r* = 0.663 (0.386 0.831) (large); *p* = 0.0001	*r* = −0.184 (−0.522 0.203) (small); *p* = 0.347	*r* = 0.472 (0.120 0.718) (moderate); *p* = 0.011	*r* = 0.229 (−0.158 0.555) (small); *p* = 0.125	*r* = 0.616 (0.315 0.804) (large); *p* = 0.315	*r* = −0.146 (−0.493 0.240) (small); *p* = 0.457	*r* = 0.434 (0.073 0.695) (moderate); *p* = 0.021	*r* = 0.245 (−0.140 0.567) (small); *p* = 0.208	*r* = 0.586 (0.272 0.787) (large); *p* = 0.001
Female (≤52 kg) *n* = 29	*r* = −0.221 (−0.549 0.166) (small); *p* = 0.259	*r* = −0.287 (−0.592 0.089) (small); *p* = 0.131	*r* = −0.115 (−0.462 0.263) (small); *p* = 0.553	*r* = 0.398 (0.037 0.667) (moderate) *p* = 0.033	*r* = 0.165 ( −0.215 0.501) (small); *p* = 0.393	*r* = 0.581 (0.2720.781) (large); *p* = 0.0001	*r* = −0.189 (−0.520 0.191) (small); *p* = 0.326	*r* = 0.335 (−0.036 0.625) (moderate); *p* = 0.076	*r* = 0.056 (−0.317 0.414) (trivial); *p* = 0.772	*r* = 0.663 (0.392 0.828) (large); *p* = 0.0001	*r* = −0.191 (−0.521 0.189) (small); *p* = 0.321	*r* = 0.353 (−0.016 0.637) (moderate); *p* = 0.060	*r* = 0.080 (−0.295 0.434) (trivial); *p* = 0.680	*r* = 0.623 (0.333 0.806) (large); *p* = 0.001
Female (≤57 kg) *n* = 25	*r* = −0.227 (−0.578 0.194) (small); *p* = 0.285	*r* = −0.275 (−0.604 0.135) (small); *p* = 0.183	*r* = −0.158 (−0.521 0.253) (small); *p* = 0.451	*r* = 0.471 (0.093 0.730) (moderate) *p* = 0.017	*r* = 0.261 (−0.149 0.595) (small); *p* = 0.207	*r* = 0.480 (0.1040.735) (moderate); *p* = 0.015	*r* = −0.161 (−0.523 0.250) (small); *p* = 0.441	*r* = 0.382 (−0.016 0.675) (moderate); *p* = 0.060	*r* = 0.157 (−0.254 0.520) (small); *p* = 0.454	*r* = 0.496 (0.125 0.745) (moderate); *p* = 0.012	*r* = −0.128 (−0.498 0.281) (small); *p* = 0.542	*r* = 0.495 (0.124 0.744) (moderate); *p* = 0.012	*r* = 0.288 (−0.121 0.613) (small); *p* = 0.163	*r* = 0.553 (0.202 0.778) (large); *p* = 0.004
Female (≤63 kg) *n* = 31	*r* = −0.310 (−0.603 0.056) (moderate); *p* = 0.095	*r* = −0.360 (−0.634–0.006) (moderate); *p* = 0.047	*r* = −0.056 (−0.305 0.402) (trivial); *p* = 0.765	*r* = 0.557 (0.253 0.761) (large) *p* = 0.001	*r* = 0.331 (−0.027 0.613) (moderate); *p* = 0.069	*r* = 0.584 (0.2890.777) (large); *p* = <0.001	*r* = 0.035 (−0.324 0.384) (trivial); *p* = 0.853	*r* = 0.471 (0.140 0.707) (moderate); *p* = 0.007	*r* = 0.237 (−0.128 0.545) (small); *p* = 0.199	*r* = 0.564 (0.262 0.765) (large); *p* = <0.001	*r* = −0.108 (−0.445 0.256) (trivial); *p* = 0.563	*r* = 0.338 (−0.019 0.618) (moderate); *p* = 0.063	*r* = 0.094 (−0.269 0.434) (trivial); *p* = 0.615	*r* = 0.561 (0.258 0.763) (large); *p* = 0.001
Female (≤70 kg) *n* = 28	*r* = −0.248 (−0.574 0.146) (small); *p* = 0.212	*r* = −0.299 (−0.605 0.083) (small); *p* = 0.122	*r* = −0.052 (−0.417 0.328) (trivial); *p* = 0.794	*r* = 0.416 (0.051 0.683) (moderate) *p* = 0.028	*r* = 0.209 (−0.178 0.540) (small); *p* = 0.286	*r* = 0.517 (0.1780.746) (large); *p* = <0.005	*r* = −0.034 (−0.402 0.344) (trivial); *p* = 0.864	*r* = 0.318 (−0.063 0.618) (moderate); *p* = 0.099	*r* = 0.191 (−0.196 0.526) (small); *p* = 0.331	*r* = 0.469 (0.116 0.717) (moderate); *p* = 0.012	*r* = −0.097 (−0.454 0.627) (trivial); *p* = 0.084	*r* = 0.332 (−0.047 0.627) (moderate); *p* = 0.084	*r* = 0.169 (−0.218 0.510) (small); *p* = 0.389	*r* = 0.455 (0.099 0.708) (moderate); *p* = 0.015
Female (≤78 kg) *n* = 24	*r* = −0.479 (−0.745 0.084) (moderate); *p* = 0.021	*r* = −0.568 (−0.791 0.214) (large); *p* = 0.004	*r* = −0.337 (−0.652 0.077) (moderate); *p* = 0.107	*r* = 0.475 (0.089 0.737) (moderate) *p* = 0.019	*r* = 0.164 (−0.256 0.532) (small); *p* = 0.164	*r* = 0.720 (0.4460.870) (very large); *p* = <0.001	*r* = −0.380 (−0.679 0.028) (moderate); *p* = 0.067	*r* = 0.075 (−0.339 0.464) (trivial); *p* = 0.729	*r* = −0.166 (−0.533 0.255) (small); *p* = 0.439	*r* = 0.577 (0.227 0.795) (large); *p* = 0.003	*r* = −0.338 (−0.652 0.076) (moderate); *p* = 0.107	*r* = 0.127 (−0.291 0.505) (small); *p* = 0.553	*r* = −0.081 (−0.469 0.333) (trivial); *p* = 0.705	*r* = 0.580 (0.231 0.797) (large); *p* = 0.003
Female (≥78 kg) *n* = 27	*r* = −0.345 (−0.646 0.049) (moderate); *p* = 0.084	*r* = −0.392 (−0.646 0.049) (moderate); *p* = 0.043	*r* = −0.265 (−0.586 0.660) (small); *p* = 0.054	*r* = 0.374 (−0.007 0.660) (moderate) *p* = 0.054	*r* = 0.087 (−0.303 0.452) (trivial); *p* = 0.666	*r* = 0.640 (0.3440.821) (large); *p* = <0.001	*r* = −0.446 (−0.706 0.080) (moderate); *p* = 0.020	*r* = 0.040 (−0.345 0.414) (trivial); *p* = 0.843	*r* = −0.233 (−0.563 0.162) (small); *p* = 0.243	*r* = 0.544 (0.207 0.766) (large); *p* = 0.003	*r* = −0.482 (−0.728 −0.125) (moderate); *p* = 0.011	*r* = 0.047 (−0.339 0.419) (trivial); *p* = 0.817	*r* = −0.238 (−0.567 0.156) (small); *p* = 0.232	*r* = 0.549 (0.214 0.769) (large); *p* = 0.003
All Female *n* = 192	*r* = −0.269 (−0.397 −0.129) (small) *p* = <0.001	*r* = −0.329 (−0.449 −0.196) (moderate) *p* = <0.001	*r* = −0.111 (−0.249 0.031) (small) *p* = 0.125	*r* = 0.454 (0.334 0.559) (moderate) *p* = <0.001	*r* = 0.204 (0.064 0.336) (small) *p* = 0.004	*r* = 0.588 (0.487 0.674) (large) *p* = <0.001	*r* = −0.186 (−0.320 −0.046) (small) *p* = 0.010	*r* = 0.309 (0.175 0.431) (small) *p* = <0.001	*r* = 0.078 (−0.065 0.217) (trivial) *p* = 0.284	*r* = 0.554 (0.448 0.645) (large) *p* = <0.001	*r* = −0.214 (−0.345 −0.075) (small) *p* = 0.003	*r* = 0.310 (0.176 0.433) (moderate) *p* = <0.001	*r* = 0.086 (−0.056 0.225) (trivial) *p* = 0.234	*r* = 0.551 (0.444 0.643) (large) *p* = <0.001
Male (≤60 kg) *n* = 23	*r* = −0.198 (−0.572 0.244) (small); *p* = 0.377	*r* = −0.249 (−0.600 0.181) (small); *p* = 0.251	*r* = −0.185 (−0.555 0.246) (small); *p* = 0.397	*r* = 0.454 (0.051 0.729) (moderate) *p* = 0.030	*r* = 0.156 (−0.274 0.534) (small); *p* = 0.478	*r* = 0.594 (0.2400.808) (very large); *p* = <0.001	*r* = −0.336 (−0.657 0.089) (moderate); *p* = 0.118	*r* = −0.012 (−0.422 0.402) (trivial); *p* = 0.958	*r* = −0.184 (−0.554 0.247) (small); *p* = 0.400	*r* = 0.557 (0.187 0.788) (large); *p* = 0.006	*r* = −0.325 (−0.650 0.101) (moderate); *p* = 0.130	*r* = 0.095 (−0.330 0.488) (trivial); *p* = 0.667	*r* = −0.117 (−0.505 0.310) (small); *p* = 0.594	*r* = 0.569 (0.205 0.795) (large); *p* = 0.005
Male (≤66 kg) *n* = 27	*r* = −0.223 (−0.562 0.180) (small); *p* = 0.273	*r* = −0.442 (−0.704 −0.075) (moderate); *p* = 0.021	*r* = −0.460 (−0.715 0.097) (moderate); *p* = 0.016	*r* = 0.256 (−0.138 0.579) (small) *p* = 0.198	*r* = −0.006 (−0.385 0.375) (trivial); *p* = 0.975	*r* = 0.579 (0.2550.786) (large); *p* = 0.002	*r* = −0.441 (−0.703 −0.073) (moderate); *p* = 0.021	*r* = 0.014 (−0.368 0.392) (trivial); *p* = 0.944	*r* = −0.194 (−0.535 0.201) (small); *p* = 0.331	*r* = 0.544 (0.206 0.766) (large); *p* = 0.003	*r* = −0.353 (−0.647 0.031) (moderate); *p* = 0.071	*r* = 0.122 (−0.270 0.480) (small); *p* = 0.544	*r* = −0.088 (−0.453 0.302) (trivial); *p* = 0.661	*r* = 0.494 (0.140 0.736) (moderate); *p* = 0.009
Male (≤73 kg) *n* = 36	*r* = −0.378 (−0.632 −0.051) (moderate); *p* = 0.025	*r* = −0.360 (−0.616 −0.036) (moderate); *p* = 0.031	*r* = −0.148 (−0.454 0.190) (small); *p* = 0.390	*r* = 0.353 (0.028 0.611) (moderate) *p* = 0.035	*r* = 0.181 (−0.157 0.481) (small); *p* = 0.291	*r* = 0.596 (0.3330.773) (large); *p* = 0.002	*r* = −0.190 (−0.488 0.148) (small); *p* = 0.266	*r* = 0.230 (−0.107 0.519) (small); *p* = 0.177	*r* = 0.083 (−0.252 0.401) (trivial); *p* = 0.629	*r* = 0.222 (−0.513 0.115) (small); *p* = 0.194	*r* = −0.222 (−0.513 0.115) (small); *p* = 0.194	*r* = 0.245 (−0.091 0.531) (small); *p* = 0.149	*r* = 0.088 (−0.248 0.405) (trivial); *p* = 0.610	*r* = 0.248 (−0.088 0.533) (small); *p* = 0.145
Male (≤81 kg) *n* = 35	*r* = −0.323 (−0.596 0.017) (moderate); *p* = 0.062	*r* = −0.338 (−0.603 −0.006) (moderate); *p* = 0.047	*r* = −0.052 (−0.378 0.286) (trivial); *p* = 0.768	*r* = 0.403 (0.081 0.649) (moderate) *p* = 0.016	*r* = 0.281 (−0.058 0.562) (small); *p* = 0.102	*r* = 0.546 (0.2600.744) (large); *p* = <0.001	*r* = −0.039 (−0.367 0.298) (trivial); *p* = 0.824	*r* = 0.284 (−0.055 0.564) (small); *p* = 0.098	*r* = 0.225 (−0.117 0.519) (small); *p* = 0.194	*r* = 0.465 (0.156 0.691) (moderate); *p* = 0.005	*r* = 0.008 (−0.327 0.340)(trivial); *p* = 0.966	*r* = 0.278 (−0.061 0.559) (small); *p* = 0.106	*r* = 0.238 (−0.104 0.529) (small); *p* = 0.169	*r* = 0.441 (0.126 0.675) (moderate); *p* = 0.008
Male (≤90 kg) *n* = 33	*r* = −0.258 (−0.557 0.099) (small); *p* = 0.153	*r* = −0.279 (−0.568 0.071) (small); *p* = 0.047	*r* = −0.075 (−0.407 0.276) (trivial); *p* = 0.680	*r* = 0.290 (−0.059 0.576) (small) *p* = 0.101	*r* = 0.147 (−0.207 0.415) (small); *p* = 0.415	*r* = 0.415 (0.0830.664) (moderate); *p* = 0.016	*r* = −0.038 (−0.376 0.310) (trivial); *p* = 0.835	*r* = 0.433 (0.105 0.676) (moderate); *p* = 0.012	*r* = 0.286 (−0.064 0.573) (small); *p* = 0.107	*r* = 0.434 (0.107 0.677) (moderate); *p* = 0.012	*r* = −0.009 (−0.351 0.335) (trivial); *p* = 0.960	*r* = 0.513 (0.206 0.728) (largel); *p* = 0.002	*r* = 0.339 (−0.005 0.611) (moderate); *p* = 0.054	*r* = 0.442 (0.117 0.682) (moderate); *p* = 0.010
Male (≤100 kg) *n* = 25	*r* = −0.634 (−0.826 −0.310) (large); *p* = <0.001	*r* = −0.472 (−0.731 0.094) (moderate); *p* = 0.017	*r* = −0.476 (−0.733 −0.100) (moderate); *p* = 0.016	*r* = 0.318 (−0.089 0.633) (moderate) *p* = 0.122	*r* = 0.033 (−0.367 0.423) (trivial); *p* = 0.876	*r* = 0.620 (0.2980.816) (large); *p* = <0.001	*r* = −0.389 (−0.680 0.007) (moderate); *p* = 0.055	*r* = 0.108 (−0.300 0.482) (trivial); *p* = 0.608	*r* = −0.065 (−0.449 0.339) (trivial); *p* = 0.758	*r* = 0.518 (0.155 0.758) (moderate); *p* = 0.008	*r* = −0.425 (−0.702 −0.036) (moderate); *p* = 0.034	*r* = 0.095 (−0.312 0.472) (trivial); *p* = 0.652	*r* = −0.096 (−0.474 0.311) (trivial); *p* = 0.646	*r* = 0.494 (0.123 0.744) (moderate); *p* = 0.012
Male (≥100 kg) *n* = 22	*r* = −0.286 (−0.639 0.166) (small); *p* = 0.209	*r* = −0.393 (−0.699 0.035) (moderate); *p* = 0.071	*r* = −0.209 (−0.579 0.233) (small); *p* = 0.351	*r* = 0.526 (0.134 0.775) (large) *p* = 0.012	*r* = 0.189 (−0.253 0.566) (small); *p* = 0.399	*r* = 0.665 (0.3380.849) (large); *p* = <0.001	*r* = −0.063 (−0.472 0.368) (trivial); *p* = 0.779	*r* = 0.436 (0.018 0.724) (moderate); *p* = 0.043	*r* = 0.267 (−0.174 0.619) (small); *p* = 0.230	*r* = 0.485 (0.080 0.753) (moderate); *p* = 0.022	*r* = −0.085 (−0.489 0.349) (trivial); *p* = 0.707	*r* = 0.437 (0.019 0.725) (moderate); *p* = 0.042	*r* = 0.220 (−0.222 0.587) (small); *p* = 0.326	*r* = 0.525 (0.133 0.755) (large); *p* = 0.012
All male *n* = 201	*r* = −0.271 (−0.396 −0.135) (small) *p* = <0.001	*r* = −0.303 (−0.424 −0.172) (small) *p* = <0.001	*r* = −0.186 (−0.317 −0.049) (small) *p* = 0.008	*r* = 0.349 (0.221 0.465) (moderate) *P* = <0.001	*r* = 0.154 (0.016 0.286) (small) *P* = 0.029	*r* = 0.549 (0.445 0.639) (large) *p* = <0.001	*r* = −0.196 (−0.326 −0.059) (small) *p* = 0.005	*r* = 0.222 (0.087 0.350) (small) *p* = 0.002	*r* = 0.077 (−0.062 0.213) (trivial) *p* = 0.277	*r* = 0.449 (0.331 0.553) (moderate) *p* = <0.001	*r* = −0.183 (−0.314 −0.046) (small) *p* = 0.009	*r* = 0.258 (0.124 0.383) (small) *p* = <0.001	*r* = 0.102 (−0.036 0.238) (trivial) *p* = 0.148	*r* = 0.445 (0.327 0.550) (moderate) *p* = <0.001
All sample *n* = 393	*r* = −0.255 (−0.346 −0.158) (trivial); *p* = 0.054	*r* = −0.296 (−0.384 −0.203) (small); *p* = 0.0001	*r* = −0.144 (−0.240 −0.046) (small); *p* = 0.004	*r* = 0.395 (0.309 0.476) (moderate) *p* = 0.0001	*r* = 0.178 (0.080 0.272) (small); *p* = <0.001	*r* = 0.564 (0.493 0.628) (large); *p* = <0.001	*r* = −0.188 (−0.282 −0.091) (small); *p* = <0.001	*r* = 0.261 (0.167 0.351) (small); *p* = <0.001	*r* = 0.077 (−0.022 0.175) (trivial); *p* = 0.125	*r* = 0.498 (0.419 0.569) (moderate); *p* = <0.001	*r* = −0.196 (−0.290 −0.100) (small); *p* = <0.001	*r* = 0.280 (0.186 0.369) (small); *p* = <0.001	*r* = 0.095 (−0.004 0.192) (trivial); *p* = 0.061	*r* = 0.495 (0.416 0.566) (moderate); *p* = <0.001

Final ranking: Position of athlete in the Olympic qualification ranking; Ranking all: Position of athlete in the World Ranking list; Competition disputed: numbers of competition disputed in the classification phase; Matches won: Matches won in the classification phase; Total matches: Total matches in the classification phase; Win%: Winning percentage during the classification phase; Competitions last year: Numbers of competitions disputed one year before the Olympic Games; Matches won last year: Matches won during the competition one year before the Olympic Games; Matches last year: Total of matches disputed one year before the Olympic Games; Win% last year: Winning percentage one year before the Olympic Games; Competition 6 months: Numbers of competition disputed six months before the Olympic Games; Matches won six months: Matches won during the competition six months before the Olympic Games; Matches six months: Total of matches disputed during six months before Olympic Games; Win% 6 months: winning percentage in the period of six months before the Olympic Games.

The ranking of the athlete, the number of competitions held during the classification phase, and the performance one year and six months prior to the Olympic Games were negatively and significantly correlated with the performance in the Olympic Games. Additionally, the winning percentage during the classification phase and the performance six months prior to the Olympic Games were positively and significantly correlated with Olympic Games performance.

Table 3 presents the equations predicting performance in the Tokyo Olympic Games based on the previous performance variables.

**Table 3:** Predictive equations of Olympic Games performance in male and female senior judo athletes.

Sex	Equation	*R*	*R* ^2^	Adjusted *R*^2^	SEE	p
Male	OGP = 6.662 + 0.575 (win %) – 0.250 (C6M)	0.600	0.361	0.354	370.08	<0.001
Female	OGP = −117.808 + 0.580 (win %) −0.146 (CLY)	0.607	0.368	0.367	364.91	<0.001
All	OGP = −29.335 + 0.572 (win%) −0.211 (CLY)	0.602	0.363	0.360	366.73	<0.001

OGP, Olympic Games points; win%, winning percentage during the classification phase; C6M, Competitions disputed six months before the Olympic Games; CLY,  Competitions disputed in the last year of the classification phase.

The independent variables, including ranking, number of competitions held, total matches, and matches won during the classification phase, percentage of wins six months prior to the Olympic Games, and the time interval between the last competition and the Olympic Games, were correlated with other independent variables (*r* values varied between 0.11 and 0.93, *p* < 0.05). Therefore, these variables were not included to avoid multicollinearity.

The variables predicting performance were different depending on sex. For male athletes, those who had the highest winning percentage and performed a lower number of competitions in the six months before event had better performance. For female athletes, a higher winning percentage and a lower number of competitions during the last year were the factors related to higher performance in the Tokyo Olympic Games. When analyzing the whole sample (males and females) similar results were found, as athletes with a higher winning percentage and lower number of competitions in the last year of the classification phase performed better in the Olympic Games.

## 4. Discussion

The main findings of the present study were that the winning rate during the classification phase and the number of competitions held in the last year before the Olympic Games could predict performance in 36% of cases. However, it is important to consider that the variables predicting performance in the Olympic Games varied between males and females judo athletes. For male athletes, the winning percentage during the classification phase and the number of competitions held six months prior to the Olympic Games predicted performance. For female athletes, the winning percentage during the classification phase and the number of competitions held during the last year of the qualifying phase (i.e., one year prior) predicted performance. Therefore, the hypothesis of the present study was partially confirmed.

These results indicate that athletes who had consistent performance during the classification phase and used strategies to limit exposure before the major competition were more successful in the Olympic Games. In addition, female athletes require a longer period of consistent performance (12 months) compared to male athletes (6 months). These results are similar to previous studies (3, 5), which reported that long-term performance (ranking position) and exposure during the year of competition (World Championship or Olympic Games) predicted the performance. However, in this present analysis, we found a higher prediction percentage (36% and 37% compared to 24% and 26% as reported by Franchini and Julio (3), and 21% and 27% as reported by Breviglieri et al. (5), for males and females, respectively). These differences may be associated with changes made to the qualification phase due to the COVID-19 pandemic. Scheduled competitions were cancelled in 2020, including the Olympic Games (7). With the start of the vaccination process, competitions were able to resume, albeit in a short period of time before the Olympic Games and in specific locations (i.e., with better control over the pandemic). As a result, some athletes may not have participated due to some factors, such as financial constraints or fear of the pandemic effects. Furthermore, the training process was hindered by lockdowns (10). Thus, a possible home advantage (12) or advantage for athletes with greater investment in the qualification phase could have influenced the predicted performance in the Olympic Games. Despite the increase in the prediction percentage, 64% of the performance could not be explained by these variables. It may be associated with the unique characteristics of the Olympic Games, which only feature the top-ranked athletes, unlike other events analyzed in the present study (3). Aspects related to technical-tactical performance of these top-ranked athletes can affect the prediction.

To explain the importance of limiting competition exposure leading up to the Olympic Games, several aspects can be highlighted: (1) Lower competition exposure can prevent opponents from studying the main actions related to the match-related performance in competition, such as variability of actions, direction of attacks, type of defenses, groundwork combat transitions and pacing strategies (13, 14, 15, 16); (2) Judo competitions have a high incidence of injuries (17), and a higher number of competitions can increase the injury rate, especially considering the short interval among competitions (e.g., 48 days between the Judo World Championship and the Olympic Games). Specifically, previous research demonstrated that the optimal interval between successive competitions should be longer than 14 weeks when the Olympic Games is considered (8). This period is important for optimal training adaptation for physical variables that are determinants in judo (18), as well as for tactical preparation for specific opponents; (3) Rapid weight loss is common in judo and is considered a psychologically demanding process for athletes (19, 20). As a suggestion for future research, the authors could consider incorporating additional variables to enhance performance prediction, such as technical-tactical aspects and explore determinants related to the home-advantage in relation to the classification phase.

## 5. Conclusion

The Olympic Games performance for male and female judo athletes can be predicted at 36% based on long-term performance and exposure during the last year of the classification phase. In other words, the main variables that explain the performance in this competition are higher performance (as indicated by their winning percentage) during the classification phase and lower, but successful, exposure close to the Olympic Games. Hence, coaches should take into account the amount of competitions in which the athletes will participate in, considering the optimal interval for success in the event.

## Data Availability

The raw data supporting the conclusions of this article will be made available by the authors, without undue reservation.
